# Breast-conserving surgery after vacuum-assisted biopsy in patients with unexpected breast cancer: is it reliable? A 5-year follow-up study

**DOI:** 10.3389/fonc.2026.1723625

**Published:** 2026-04-15

**Authors:** Hong-Yan Fang, Wen-Ting Zheng, Yang-Jun Cai, Fang-Suan Zhu, Lin-Yi Wang, Jia-Xiang An, Bo-Jian Xie, Zeng-Gui Wu

**Affiliations:** Department of Surgical Oncology, Taizhou Hospital of Zhejiang Province affiliated to Wenzhou Medical University, Linhai, Zhejiang, China

**Keywords:** BI-RADS, breast cancer, breast-conserving surgery (BCS), mastectomy, vacuum-assisted breast biopsy (VABB)

## Abstract

**Background:**

Vacuum-assisted breast biopsy (VABB) is commonly used for complete resection of benign tumors and diagnostic biopsy of suspected malignant tumors in China. Post-procedural hematoma formation may potentially facilitate tumor cell dissemination into surrounding tissues. When pathological analysis confirms malignancy, does subsequent breast-conserving surgery (BCS) affect long-term prognosis? This study aims to use five-year follow-up data to investigate these issues.

**Methods:**

All patients diagnosed with malignancy following VABB between January 2016 and January 2020 were included in this study. These patients were subsequently stratified into two cohorts based on their selected surgical approach (BCS vs. mastectomy). Clinical and pathological characteristics, comprehensive treatment regimens, and recurrence rates were systematically compared between the groups.

**Results:**

A cohort of 124 patients with BI-RADS category 3 or 4a lesions, diagnosed with incidental breast malignancies, was enrolled. The breast-conserving surgery (BCS) group comprised 64 patients (51.6%), of whom 6 demonstrated positive resection margins (9.4%) and 2 ultimately underwent conversion to total mastectomy. Preoperative clinical parameters showed no significant intergroup differences except for tumor location. During follow-up, 8 patients developed ipsilateral recurrence or contralateral new primaries, with 3 subsequent cancer-related deaths; however, no statistically significant differences were observed between the surgical groups.

**Conclusions:**

Hematoma and residual cavities may appear after VABB in unexpected breast cancer, potentially affecting surgical visualization and raising concerns about tumor cell extravasation. However, as long as the cavity is completely removed to ensure negative surgical margins, further BCS does not adversely affect patient prognosis.

## Introduction

Breast cancer is the most prevalent cancer among women and has become the leading cause of cancer death in women over the age of 45 (1). Advances in medical imaging technologies (including high-resolution ultrasound, mammography, and magnetic resonance imaging) have driven a significant increase in the detection rate of early-stage breast cancer in recent decades (2). Compared with traditional open surgical biopsy, which causes more trauma and affects aesthetics, minimally invasive biopsies are now more commonly used for the early diagnosis of breast lesions because of minimal discomfort and barely perceptible scars. Common diagnostic modalities include fine-needle aspiration biopsy (FNAB), core-needle biopsy (CNB), and vacuum-assisted breast biopsy (VABB). For small or even nonpalpable tumors, particularly in patients with BI-RADS category 3 or 4a lesions demonstrating low malignant potential, ultrasound-guided vacuum-assisted biopsy currently represents the most important method in the diagnosis of early-stage breast cancer due to the large amount of tissue removed and its high accuracy and low false negative rate compared with core needle biopsy (CNB) (3).

Given the advantages of VABB in achieving complete tumor resection with minimal surrounding tissue trauma and superior cosmetic outcomes, the pioneering Mammotome^®^ system, the first FDA approved vacuum-assisted biopsy device, was initially indicated for excising benign breast lesions measuring ≤ 3cm in maximal diameter (4). In China, women’s breast tissue is relatively dense, making mammography less sensitive (5). Breast magnetic resonance imaging (MRI) is not widely used due to its high cost and long waiting times, so most patients rely on breast ultrasound for diagnosis. Interobserver variability in sonographers’ interpretation of subtle malignant features in breast lesions is predominantly reflected through inconsistencies in BI-RADS categorization documented in ultrasound reports. That is to say, for breast cancer that shows benign features on ultrasound images, it may be classified into a lower ultrasound categorization, resulting in patients deemed benign preoperatively being eventually diagnosed as malignant. According to prior studies, the rate of tumor residue after VABB is substantial, with up to 70-80%. Once malignancy is confirmed, secondary open surgery must be performed. The most frequent complications associated with VABB include intraprocedural hemorrhage and post-interventional hematoma formation. These sequelae may compromise surgical margin assessment and theoretically increase risks of iatrogenic tumor cell dissemination into adjacent breast parenchyma. Thus, it remains unclear whether this situation affects the choice of surgical methods, especially the safety of breast-conserving surgery.

So far, there have been no reports on the safety and prognosis of breast-conserving surgery for unexpected early breast cancer diagnosed by VABB. Our research focuses on exploring the success rates, influencing factors and survival analysis of breast-conserving surgery in breast cancer patients who were incidentally diagnosed after BI-RADS category 3 or 4a and were considered benign before surgery.

## Methods

**Figure 1:**
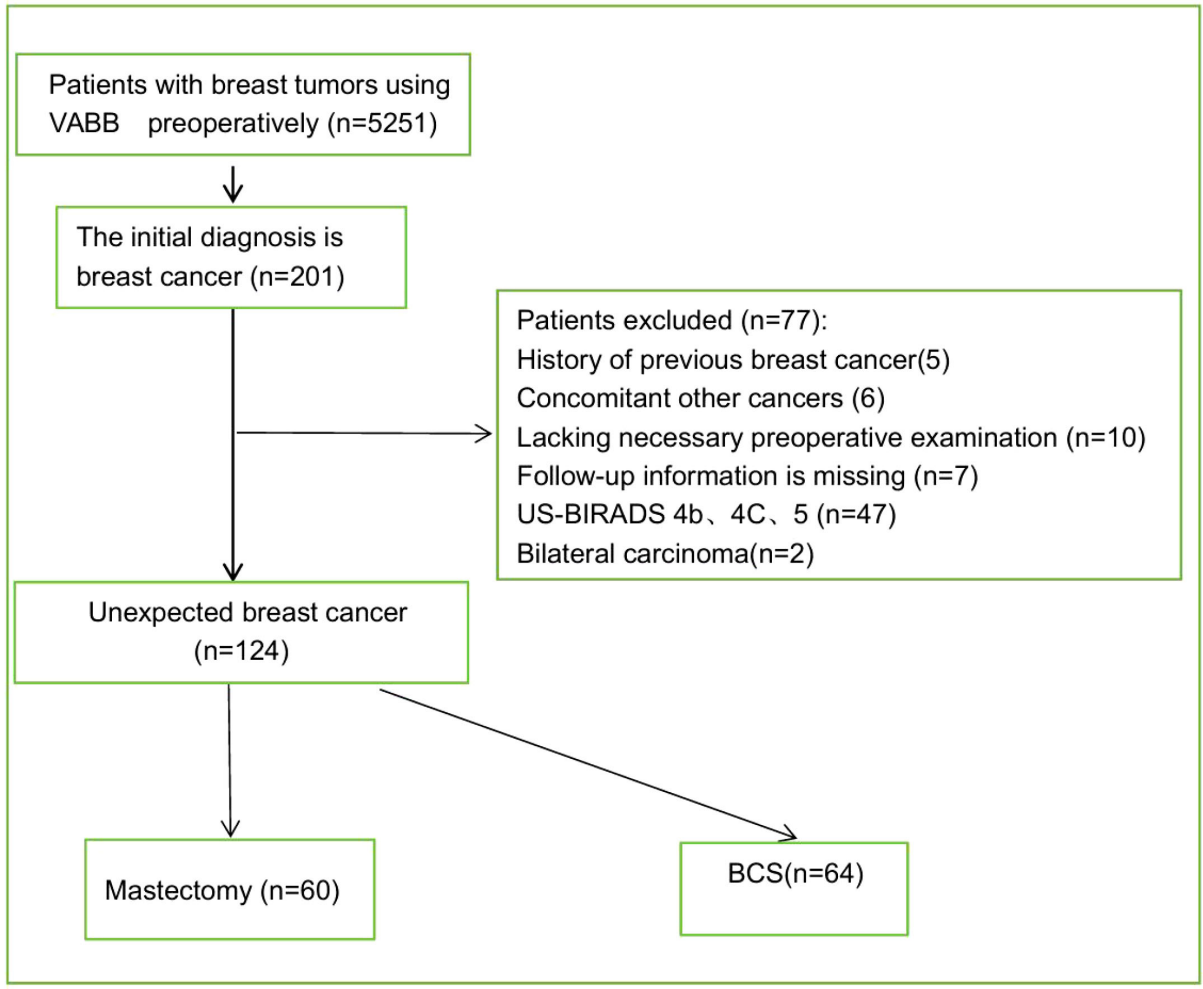
A flowchart showing the inclusion and exclusion criteria and selection steps.

Based on our retrospective database, we identified all patients diagnosed with breast cancer following VABB at The Affiliated Taizhou Hospital, Wenzhou Medical University, between January 2016 and January 2020. Of the 5251 patients who underwent VABB, 201 patients were diagnosed with breast cancer. The exclusion criteria included the following: a history of previous breast cancer, concomitant other cancers, lack of necessary preoperative examination, missing follow-up information, bilateral carcinoma, and patients with BI-RADS category 4b, 4c, or 5 lesions who underwent VABB as a diagnostic intervention, reflecting their elevated malignant potential (category 4b: 10-50%, 4c: 50-95%, 5: >95% per ACR guidelines). The inclusion and exclusion criteria, along with the selection procedure, are detailed in the flowchart (**Figure 1**). Ultimately, 124 patients with unexpected breast cancer diagnosed after VABB were enrolled in our study. All patients underwent preoperative ultrasonography prior to undergoing a VABB, and the results were categorized according to the American College of Radiology Breast Imaging Reporting and Data System (BI-RADS) (6). We meticulously recorded the size, location, and number of tumors removed in each patient. According to the location of the tumors, we categorized them into the outer upper quadrant, inner upper quadrant, outer lower quadrant, inner lower quadrant, and central area (less than 1cm from the nipple). Vacuum-assisted biopsies were performed using the Mammotome^®^ system. If malignancy was confirmed by Intraoperative frozen pathology examination during the biopsy, immediate further expansion surgery was performed. If malignancy was confirmed by routine postoperative paraffin pathology, delayed expansion surgery was performed within 2 weeks. The presence of two or more malignant tumors was considered multifocal. Other preoperative clinical data included the patients’ age at diagnosis.

For patients who intended to undergone breast-conserving therapy and met the criteria for procedure, breast-conserving surgery was preferred whenever possible. The decision to perform axillary dissection was based on the results of the sentinel lymph node biopsy and the surgeon’s discretion. The results of intraoperative frozen pathology and postoperative routine paraffin pathology were reevaluated and diagnosed by two senior pathologists. Histological types were categorized as carcinoma *in situ*, invasive carcinoma of no special type (NST), and invasive carcinoma of special type. By integrating immunohistochemical determinations of estrogen receptor (ER), progesterone receptor (PR), HER2 status, and Ki-67 proliferation index, patients were classified into the following four molecular subtypes: Luminal A, Luminal B, HER2-enriched, and TNBC, in accordance with the criteria established by the St Gallen International Expert Consensus (7). Breast cancer staging was conducted using the TNM classification system, incorporating tumor size (T category) and axillary lymph node involvement (N category) (8). The presence of residual tumor in the cavity was determined based on postoperative pathological results. Both the VABB and the breast cancer surgery were performed during the same hospitalization.

Breast cancer is a systemic disease. All patients received postoperative therapies, including chemotherapy, radiotherapy, endocrine therapy, and targeted therapy, based on pathological findings and adherence to the National Comprehensive Cancer Network (NCCN) guidelines. Following the operation, each patient underwent periodic examinations, including imaging modalities (ultrasound, high-resolution CT, MRI) and serum tumor marker assays, to assess recurrence or metastasis. Based on recurrence patterns, patients were categorized into three groups: recurrence-free, disease-progression, and deceased.The primary endpoint was disease-free survival (DFS): time from surgery to first ipsilateral recurrence (invasive or *in situ*), distant metastasis (beyond locoregional nodes), contralateral new primary (pathologically confirmed), or death. Overall survival (OS) was measured from surgery to death from any cause. Due to the limited number of outcome events, formal time-to-event modeling was not performed, and survival outcomes were descriptively compared between groups.

### Statistical analysis

We applied SPSS version 26 software for statistical analysis to explore differences between groups regarding specific variables. The t-test was used to examine differences in continuous variables, while the chi-squared test or Fisher’s exact test was used for categorical variables. Normal data are presented as mean ± standard deviation, and non-normal data are presented as median and interquartile range. A p-value of less than 0.05 was considered statistically significant.

## Results

Eventually, a total of 124 patients with unexpected breast cancer were enrolled in our study. Before VABB, all tumors were considered benign. VABB was indicated in cases of recent tumor enlargement or when patients raised concerns about possible malignancy. Of these, 64 patients chose breast-conserving surgery following a confirmed diagnosis of malignant tumors, while the remaining 60 patients proceeded directly to total mastectomy due to ineligibility for breast-conserving surgery. The median age of the breast-conserving group was 47.23 ± 9.29, slightly younger than that of the total mastectomy group (49.33 ± 8.51). All tumors measured <25 mm. Tumors ≤20 mm accounted for 95.0% and 95.3% of cases in the breast-conserving and total mastectomy groups, respectively, corresponding to the T1 category per TNM staging. We observed that 75.0% (48/60) of cases in the breast-conserving group were classified as BI-RADS category 4a, comparable to 70.0% (42/60) of cases in the total mastectomy group. There was no significant difference between the two groups in terms of tumor location on the left or right sides; however, a significant difference was observed in the quadrant distribution of tumors between the two groups (p = 0.005). The breast-conserving group had more tumors in the outer upper quadrants, whereas all tumors in the central area underwent total mastectomy. Multifocality was defined as the presence of at least two malignant foci within the ipsilateral breast. Although the difference did not reach statistical significance, total mastectomy was more commonly performed in these cases in our study. The majority of patients were confirmed to have malignant tumors during intraoperative frozen pathology examination and subsequently underwent additional surgical procedures. There was no significant difference between the two groups (85.0%vs84.4%, p=0.923). Overall, the breast-conserving and mastectomy groups were well-balanced in terms of baseline clinical characteristics. Detailed information is presented in **Table 1**.

**Table 1:** Preoperative characteristics of breast cancer patients undergoing VABB.

Characteristic	Surgical method		
	Mastectomy (60)	BCS (64)	P
Age (years)	49.33 ± 8.51 (33-79)	47.23 ± 9.29 (29-77)	0.193
Tumor size			0.991
≤10mm	20 (33.3%)	22 (34.4%)	
10-20mm	37 (61.7%)	39 (60.9%)	
>20mm	3 (5.0%)	3 (4.7%)	
BI-RADS			0.533
3	18 (30.0%)	16 (25.0%)	
4A	42 (70.0%)	48 (75.0%)	
Lesion side			0.871
Left	31 (51.7%)	34 (53.1%)	
Right	29 (48.3%)	30 (46.9%)	
Which quadrant			0.005
Outer upper	21 (35.0%)	40 (62.5%)	
Inner upper	18 (30.0%)	9 (14.1%)	
Outer lower	10 (16.7%)	10 (15.6%)	
Inner lower	5 (8.3%)	5 (7.8%)	
Central district	6 (10.0%)	0 (0)	
Multifocality			0.233
YES	6 (10.0%)	2 (3.1%)	
NO	54 (90.0%)	62 (96.9%)	
Timing of Surgery			0.923
Immediate	51 (85.0%)	54 (84.4%)	
Delay	9 (15.0%)	10 (15.6%)	

BCS, breast-conserving surgery.

Multifocality, At least two malignant lesions in Ipsilateral breast

Timing of Surgery, Timing of Surgery directly after biopsy or delayed

Routine postoperative pathological results revealed that the proportion of tumor residual was higher in the mastectomy group (81.7% vs73.4%), although the difference was not statistically significant (p=0.273). Axillary dissection was performed only after a positive sentinel lymph node biopsy was detected. The prevalence of positive axillary lymph nodes was not significantly different between the two groups (18.3% vs. 10.9%, p=0.243). Ki-67 is a biomarker reflecting tumor proliferation index. The proportion of tumors with Ki-67 expression >14% was higher in the breast-conserving group (67.2%) than in the mastectomy group (58.3%), though this difference was not statistically significant (p=0.308). There was no significant difference in molecular subtype between the two groups of patients(p=0.750). The most common subtype was Luminal B, followed by Luminal A and HER2-enriched subtypes; the least prevalent was triple-negative breast cancer (TNBC). Based on pathological classification, carcinoma *in situ* was significantly more prevalent in the mastectomy group (25.0% vs. 9.4%; p=0.048), potentially accounting for the differences in TNM stages between the two groups (p=0.026). The proportion of TNM stage 0 was higher in the total mastectomy group. Most patients in both groups received chemotherapy after surgery (63.3% vs. 67.2%, p=0.652). In the breast-conserving group, only 5 patients did not receive radiotherapy: 2 were older than 70 years, and 3 declined radiotherapy. In the mastectomy group, radiotherapy was considered only for lymph node metastasis, resulting in a significantly lower rate compared to the breast-conserving group (18.3% vs. 92.2%, p < 0.001). There were no significant differences in postoperative factors between the two groups, as detailed in **Table 2**.

**Table 2:** Postoperative factors and recurrence rate between the mastectomy group and BCS group.

Variables	Surgical method		
	Mastectomy (60)	BCS (64)	P
Tumor residual			0.273
YES	49 (81.7%)	47 (73.4%)	
NO	11 (18.3%)	17 (26.6%)	
Molecular subtype			0.750
Luminal A	21 (35.0%)	23 (35.9%)	
Luminal B	23 (38.3%)	25 (39.1%)	
HER2-enriched	11 (18.3%)	8 (12.5%)	
TNBC	5 (8.3%)	8 (12.5%)	
N stage			0.243
N0	49 (81.7%)	57 (89.1%)	
N1	11 (18.3%)	7 (10.9%)	
Ki-67			0.308
≤14%	25(41.7%)	21(32.8%)	
>14%	35 (58.3%)	43 (67.2%)	
TNM Stage			0.026
0	15 (25.0%)	6 (9.4%)	
IA	30 (50.0%)	48 (75.0%)	
IIA	12 (20.0%)	8 (12.5%)	
IIB	1 (1.7%)	2 (3.1%)	
IIIA	2 (3.3%)	0 (0)	
Histology			0.048
Carcinoma in situ	15 (25.0%)	6 (9.4%)	
Invasive non-special cancer	42 (70.0%)	56 (87.5%)	
Invasive special cancer	3 (5.0%)	2 (3.1%)	
Adjuvant therapy			
Chemotherapy	38 (63.3%)	43 (67.2%)	0.652
Radiation	11 (18.3%)	59 (92.2%)	p<0.001
Follow-up (months)	79.83 ± 14.07	83.19 ± 14.80	0.198
Recurrence or metastasis	2 (3.3%)	3 (4.7%)	1.0
Contralateral new cancer	2 (3.3%)	1 (1.6%)	1.0
Died of breast cancer	1 (1.7%)	2 (3.1%)	1.0
5-year specific survival rate	98.3%	96.9%	1.0

Tumor residual, residual cavity with tumor after VABB.

Specificity, Special types of breast cancer, such as mucinous carcinoma, neuroendocrine carcinoma, and tubular carcinoma.

Among the 64 patients in the breast-conserving group, 6 patients had positive surgical margins during the first examination, and 2 patients were subsequently converted to mastectomy due to positive re-resection or multiple positive margin status. The remaining 4 patients achieved negative surgical margins after extended resection. The final success rate of breast-conserving was 96.8%. After analyzing all factors that may be related to positive margin status, including size, BI-RADS grade, and tumor residue, we found that only the age difference was statistically significant between the two groups (39.33 ± 7.06 vs48.05 ± 9.16,p=0.027). A younger age was associated with a higher likelihood of positive surgical margins. Detailed information is presented in **Table 3**.

**Table 3:** Clinical characteristics of patients with different surgical margin properties in the BCS group.

Characteristic	Surgical margin		
	Positive (6)	Negative (58)	P
age cohort	39.33 ± 7.06	48.05 ± 9.16	0.027
Tumor size			
≤10mm	0	22 (37.9%)	0.089
10-20mm	5 (83.3%)	34 (58.6%)	
>20mm	1 (16.7%)	2 (3.5%)	
BI-RADS			1.0
3	1 (16.7%)	15 (25.9%)	
4A	5 (83.3%)	43 (74.1%)	
Multifocality			0.441
YES	1 (16.7%)	1 (1.7%)	
NO	5 (83.3%)	57 (98.3%)	
Duration of surgery			1.0
Immediate	5 (83.3%)	49 (84.5%)	
Delay	1 (16.7%)	9 (15.5%)	
Tumor residual			0.927
YES	5 (83.3%)	42 (72.4%)	
NO	1 (16.7%)	16 (27.6%)	

To date, the median follow-up time for patients in both groups has exceeded 5 years. In the breast-conserving group, 1 patient developed contralateral cancer, 3 patients experienced local recurrence or metastasis, and ultimately, 2 patients died. In the mastectomy group, 2 patients developed contralateral cancer, 2 patients experienced local recurrence or metastasis, and 1 patient died. There were no significant differences between the two groups. Detailed information is presented in **Table 4**.

**Table 4:** Clinical and pathological information of 8 patients with contralateral new cancer,metastasis or recurrence.

Case no	Surgical method	Tumor residual	Molecular subtype	TNM Stage	Recurrence form	Death	DFS (month)	OS (month)
22	BCS	YES	TNBC	pT1N0M0	contralateral new cancer	NO	14	/
28	mastectomy	YES	HER2-enriched	pT1N1M0	metastasis	Yes	37	44
47	BCS	YES	Luminal B	pT1N1M0	Ipsilateral breast recurrence	NO	23	/
75	mastectomy	NO	Luminal B	pT1N1M0	metastasis	NO	52	/
77	mastectomy	YES	Luminal A	pT1N0M0	contralateral new cancer	NO	52	/
88	BCS	NO	TNBC	pT1N0M0	metastasis	YES	13	59
97	BCS	YES	TNBC	pT1N0M0	metastasis	YES	8	13
106	mastectomy	YES	Luminal A	pT1N0M0	contralateral new cancer	NO	49	/

A flowchart showing the inclusion and exclusion criteria and selection steps.

## Discussion

Mammotome^®^ system, a type of vacuum-assisted biopsy, was first developed and used for diagnosing suspicious breast tumors in 1995 (9). This system is relatively easy to operate and results in minimal postoperative trauma and less noticeable incision scars. It is primarily used for the complete resection of benign tumors in Europe and the United States (4, 10). According to Chinese guidelines, VABB is widely used for the complete resection of breast tumors with low-risk factors for breast cancer, specifically those categorized as BI-RADS 3 or 4a on ultrasound (11). Despite the low likelihood of a malignant diagnosis following VABB (12), some patients unexpectedly receive a malignant diagnosis.

In China, women’s breast tissue is relatively dense, rendering mammography less sensitive to breast cancer (5, 13). Therefore, breast cancer screening mainly relies on ultrasound. With the increasing popularization of breast cancer prevention and screening, many nodules with benign features have been detected through ultrasound. Among patients with low-risk breast lesions, those exhibiting recent tumor enlargement or expressing significant anxiety may require surgical intervention, and VABB is typically the preferred minimally invasive approach. Thus, some very early-stage breast cancers are detected. In our study, the proportion of unexpected breast cancer was approximately 2.4% (124/5251), similar to previous studies (14–16). Among the 124 cancers, 98.4% (122/124) were classified as stage 0-II.

Once malignancy is diagnosed after VABB, further surgery is necessary. The chief surgeon will determine the surgical approach in adherence to the NCCN Guidelines pertaining to indications and contraindications for breast-conserving surgery, while also incorporating the patient’s preferences (17). Hematomas after VABB are often significant, tumor cells may theoretically exude into surrounding tissues through the bloodstream, which potentially violates the no-touch principle. Does this affect the surgeon’s choice of surgical methods? Our research shows that the proportion of breast-conserving surgery remains high, at 51.6% (64/124). This is similar to the results of Teng R et al (18) and higher than with other biopsy methods. This may be attributed to the fact that the patients were in the very early stages.

As our research results show, BCS is more frequently preferred for tumors located in the outer and upper quadrants due to the relatively large tissue volume, which can be filled through the transfer of fascial tissue flaps (19). There is relatively little breast tissue in the inner quadrant, which can significantly affect appearance when further enlarged surgery is performed. In the past, BCS required larger surgical margins, so when the tumor was close to the nipple and located in the central area, total mastectomy was usually chosen. Surgical margins for BCS are increasingly being narrowed. Provided that the resection margins are histologically negative (i.e., ‘no ink on tumor’), BCS remains a feasible option (20). Moreover, central breast-conserving surgery (e.g., for centrally located tumors) is gaining broader clinical acceptance (21, 22). Patients with multifocal tumors are more likely to choose mastectomy because different tumors may be located in different quadrants, which can affect both appearance and safety. In our cohort, the majority of patients underwent immediate re-excision after intraoperative frozen section analysis confirmed margin involvement. The recent surgical cavity facilitated en bloc resection with clear margins by excising the cavity and adjacent normal tissue. By contrast, delayed reoperation (≥4 weeks post-initial surgery) posed technical difficulties in identifying the residual cavity, often requiring extended resection to obtain histologically negative margins. Thus, accurately locating the residual cavity is crucial. The mastectomy group had a higher proportion of patients with carcinoma *in situ* than the BCS group, which may be due to the fact that carcinoma *in situ* tends to be more extensive and multifocal (23, 24). Factors such as tumor size and age did not affect the choice of surgical method, probably because all patients had small tumors and were younger.

Among the 64 patients who underwent BCS, 9.4% (6/64) had a positive margin status at the initial examination. This ratio is similar to those reported in previous studies (16, 25, 26), but it remains high. In centers where intraoperative frozen section analysis is unavailable, patients may require reoperation to obtain histologically negative margins. This can result in significant financial and physical burdens for patients. Kong Y et al. (16) found that margin positivity was correlated with tumor residual (P = 0.044) but not with hematoma. Our study did not find similar results. Among all risk factors, we found that age was associated with the positive rate of surgical margins in BCS. Younger patients typically have high expectations for both breast conservation and appearance. As a result, to achieve a better cosmetic outcome, less surrounding tissue is removed during the primary operation, which can increase the likelihood of positive margins. We are preparing to use a device similar to a urinary catheter to better locate the original tumor cavity during surgery. This approach aims to minimize the removal of healthy breast tissue while ensuring complete tumor excision, thereby achieving a better cosmetic result.

After a median follow-up of at least 5 years, few patients in both groups had recurrence or metastasis. When performing BCS, we removed extensive tissue and repeatedly flushed the area with distilled water during the procedure. There were no statistical differences in recurrence rates or mortality between the two groups, indicating that BCS after VABB has no effect on these outcomes. Upon in-depth study of patients who experienced recurrence or death, we found that these patients had poor molecular subtypes, such as HER2-enriched, triple-negative, or had axillary lymph node metastasis detected during the initial surgery. The prognosis of patients is primarily related to the nature of the tumor itself.

Previous retrospective studies evaluating vacuum-assisted biopsy in breast cancer have largely relied on descriptive or unadjusted analyses, particularly when oncologic events were limited. Such approaches reflect the constraints of observational data rather than a lack of methodological rigor.In our cohort, baseline differences in TNM stage, carcinoma *in situ* proportion, and radiotherapy exposure likely represent variations in tumor characteristics and treatment selection rather than the direct effect of biopsy modality. Given the non-randomized design, residual confounding cannot be excluded.

Our research has certain clinical value, but it also has some limitations. Our sample size is small, and the study is single-centered. We need to include more patients from multiple centers in the study. The patients we selected all had early-stages tumors, so the risk of recurrence was low. Moreover, the proportion of patients with HR+ was high, and the recurrence time was relatively late, so our follow-up time may not be sufficient.The small number of recurrence and death events substantially limits statistical power and precludes robust survival modeling. Therefore, the findings should be interpreted cautiously.

## Conclusion

In short, for unexpected breast cancer diagnosed after vacuum-assisted biopsy, there is theoretically a possibility that the tumor cell may spread into surrounding tissue, but the actual risk is negligible. In this cohort, BCS after VABB was not associated with an apparent increase in recurrence during follow-up. However, it is necessary to completely remove the tumor tissue in one procedure to achieve negative surgical margins and avoid secondary surgery, especially in young women.

## Data Availability

The datasets presented in this study can be found in online repositories. The names of the repository/repositories and accession number(s) can be found in the article/supplementary material.
